# Detection of Patients at Risk of Multidrug-Resistant Enterobacteriaceae Infection Using Graph Neural Networks: A Retrospective Study

**DOI:** 10.34133/hds.0099

**Published:** 2023-11-20

**Authors:** Racha Gouareb, Alban Bornet, Dimitrios Proios, Sónia Gonçalves Pereira, Douglas Teodoro

**Affiliations:** ^1^ Department of Radiology and Medical Informatics, University of Geneva, Geneva, Switzerland.; ^2^ HES-SO University of Applied Arts Sciences and Arts of Western Switzerland, Geneva, Switzerland.; ^3^ Center for Innovative Care and Health Technology, Polytechnic of Leiria, Leiria, Portugal.; ^4^ Swiss Institute of Bioinformatics, Lausanne, Switzerland.

## Abstract

**Background**: While Enterobacteriaceae bacteria are commonly found in the healthy human gut, their colonization of other body parts can potentially evolve into serious infections and health threats. We investigate a graph-based machine learning model to predict risks of inpatient colonization by multidrug-resistant (MDR) Enterobacteriaceae. **Methods:** Colonization prediction was defined as a binary task, where the goal is to predict whether a patient is colonized by MDR Enterobacteriaceae in an undesirable body part during their hospital stay. To capture topological features, interactions among patients and healthcare workers were modeled using a graph structure, where patients are described by nodes and their interactions are described by edges. Then, a graph neural network (GNN) model was trained to learn colonization patterns from the patient network enriched with clinical and spatiotemporal features. **Results:** The GNN model achieves performance between 0.91 and 0.96 area under the receiver operating characteristic curve (AUROC) when trained in inductive and transductive settings, respectively, up to 8% above a logistic regression baseline (0.88). Comparing network topologies, the configuration considering ward-related edges (0.91 inductive, 0.96 transductive) outperforms the configurations considering caregiver-related edges (0.88, 0.89) and both types of edges (0.90, 0.94). For the top 3 most prevalent MDR Enterobacteriaceae, the AUROC varies from 0.94 for *Citrobacter freundii* up to 0.98 for *Enterobacter cloacae* using the best-performing GNN model. **Conclusion:** Topological features via graph modeling improve the performance of machine learning models for Enterobacteriaceae colonization prediction. GNNs could be used to support infection prevention and control programs to detect patients at risk of colonization by MDR Enterobacteriaceae and other bacteria families.

## Introduction

Healthcare-associated infection (HAI) is a severe health problem for patients, health professionals, and visitors in a healthcare facility [1,2]. The World Health Organization estimates that 1 in every 10 patients develops an HAI [3], and in US hospitals alone, the Centers for Disease Control and Prevention estimate that HAIs account for 1.7 million infections and 99,000 associated deaths each year [4]. Among these infections, more than one-third are caused by Enterobacteriaceae [5], a family of bacteria that includes the most prevalent human pathogenic species and leading causes of nosocomial infections, such as *Escherichia coli*, *Salmonella enterica*, and *Klebsiella pneumoniae*. Given that these infections are acquired in environments under high antimicrobial pressure, they are often caused by antimicrobial-resistant (AMR) and multidrug-resistant (MDR) bacteria. MDR Enterobacteriaceae infections have augmented drastically over the last 2 decades, especially with the rise of carbapenemase-producing Enterobacteriaceae [6]. These pathogens are able to resist not only the action of all available beta-lactams (except aztreonam), but also other available antimicrobial classes like fluoroquinolones and aminoglycosides, leaving physicians with few treatment options [7]. This leads to more expensive treatments, longer hospital stays, increased risk of complication, and higher risk of death [8].

The continuous rise of these pathogens in healthcare settings is multifactorial, main contributors being their ability to spread and persist in the environment and asymptomatically in patients, as well as healthcare workers and utilities [9]. The risk of colonization, subsequent infection, and mortality due to Enterobacteriaceae increases exponentially with age, health history, and length of hospital stay [10]. Colonization can be defined as the asymptomatic presence of a pathogen in the human body. It is the first step toward an overt disease of the colonized patient, with more or less severity, and also one of the main contributors to infection outbreaks in healthcare settings [11]. Indeed, some studies showed that between 36% and 39% of patients colonized by AMR Enterobacteriaceae develop a subsequent infection [12,13]. Asymptomatic colonizations, especially by MDR bacteria, pose a prominent public health problem as the pathogen that the colonized patient carries can inadvertently be transmitted to other patients, who can become infected, with increased risk of complications, and even death [6]. Infection prevention and control (IPC) programs provide critical measures for preventing disease transmission in healthcare settings, with the potential to lower HAI rates by at least 30% [14], which is sometimes the only solution to prevent and avoid these MDR colonizations and infections.

Leveraging the availability of large-scale healthcare data [15–17], routinely collected and stored in electronic health records (EHRs), machine learning models have been proposed for the early detection of patients at risk of infection and to support IPC programs [18–22]. Classic machine learning methods, such as decision trees and random forest, have demonstrated good performance to predict patients at risk of HAI [23–26]. For methicillin-resistant *Staphylococcus aureus* [23] and *Clostridioides difficile* [26], these algorithms were shown to provide warnings as early as 5 days before diagnosis. Machine learning methods for colonization prediction were also explored in very recent studies [27–29]. Tree-based machine learning methods, such as decision trees, random forest, and extreme gradient boosting, achieved sensitivity and specificity above 80% for detecting MDR species from different pathogenic families [28], while the use of spatiotemporal features to identify patients colonized by vancomycin-resistant *Enterococcus* resulted in area under the receiver operating characteristic curve (AUROC) performance above 88% [27].

While classic machine learning models and hand-crafted features might show effective results in limited use cases, they often fail to generalize to large-scale and longitudinal EHR data [30,31]. Another limitation of previous approaches for Enterobacteriaceae colonization prediction is that key interactions between patients and healthcare workers are neglected, hindering their application to complex care networks. To address these gaps, we propose a deep-learning approach based on a graph neural network (GNN) architecture [32]. This approach aims to incorporate interactions between patients and healthcare workers, inside and outside the wards, as well as other clinical and spatiotemporal features, to predict the risk of Enterobacteriaceae colonization for inpatients. Our models were trained and evaluated using the Medical Information Mart for Intensive Care (MIMIC-III) dataset [33] and compared with classic machine learning baselines. Interestingly, GNN models provide stronger predictive performance for early detection of antimicrobial-sensitive (AMS), AMR, and MDR Enterobacteriaceae, compared to baseline models trained with or without patient network information. Our main contributions can be summarized as follows:

• We propose a graph-based colonization model that considers spatiotemporal features in addition to demographic and clinical condition. To avoid adding biases to the model due to information leakage, we do not use antimicrobial information.
• We design GNN models for colonization prediction that learn transmission network patterns from spatiotemporal and patient data. Different network configurations and transmission paths are proposed and evaluated.
• We evaluate our model against classic state-of-the-art machine learning baselines and show that it achieves superior performance, both when baseline models access network information via node2vec [34] embeddings, or not. We also conduct an explainability study to demonstrate the capacity of the model to automatically identify features associated with colonization risk factors.
• There have been many studies investigating HAI prediction. To the best of our knowledge, this is the first attempt to explore the problem of predicting risks of Enterobacteriaceae colonization for undesirable body parts using GNNs and provide data-driven hypothesis for transmission.

## Methods

### Study design and data sources

To train and evaluate our colonization risk prediction models, we used laboratory, clinical, and administrative data from patients who stayed in critical care units of the Beth Israel Deaconess Medical Center (Massachusetts, USA). These data were recorded between 2001 and 2012 and made publicly available through the MIMIC-III dataset [33]. MIMIC-III is a freely available and deidentified healthcare dataset that consists of 26 tables and includes static and dynamic patient information, such as demographics, medical history and records, clinical measures, laboratory tests, and interventions. The database contains data from 46,520 unique patients aged 16 years or older and associated to 58,976 admissions. Patients can be admitted to the hospital more than once and moved between 50 different wards and 7 care units during their stays. Additionally, activities from 7,567 unique healthcare workers—a nurse or a medical doctor—are recorded.

**Fig. 1.**
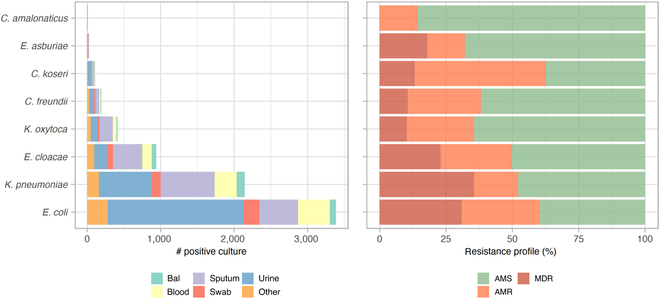
Frequency of positive culture and resistant profile for each Enterobacteriaceae family member. Species with less than 5 positive cultures are not shown. Bal, bronchoalveolar lavage.

In the MIMIC-III dataset, 10% of inpatients had a positive result for Enterobacteriaceae screen. In total, 14 different bacterial species of the Enterobacteriaceae family were found from a total of 30 unique specimen types collected from inpatients. Figure 1 shows their distribution for different sample types (Fig. 1, left) and different resistant profiles (Fig. 1, right). *E. coli* was the most frequently found species in positive cultures (50%), while *Citrobacter amalonaticus* and *Salmonella enterica* (not shown) were rarely found. A bacterial isolate was considered AMR if it showed resistance to at least one agent in only 1 or 2 antimicrobial categories, and MDR if resistant to at least one agent in 3 or more antimicrobial categories [35]. Otherwise, it was classified as AMS. As shown in Fig. 1, *Citrobacter koseri*, *E. coli*, and *K. pneumoniae* were the species with the highest levels of resistance (>50%). Both *E. coli* and *K. pneumoniae* showed MDR profiles in more than 25% of cases.

**Fig. 2.**
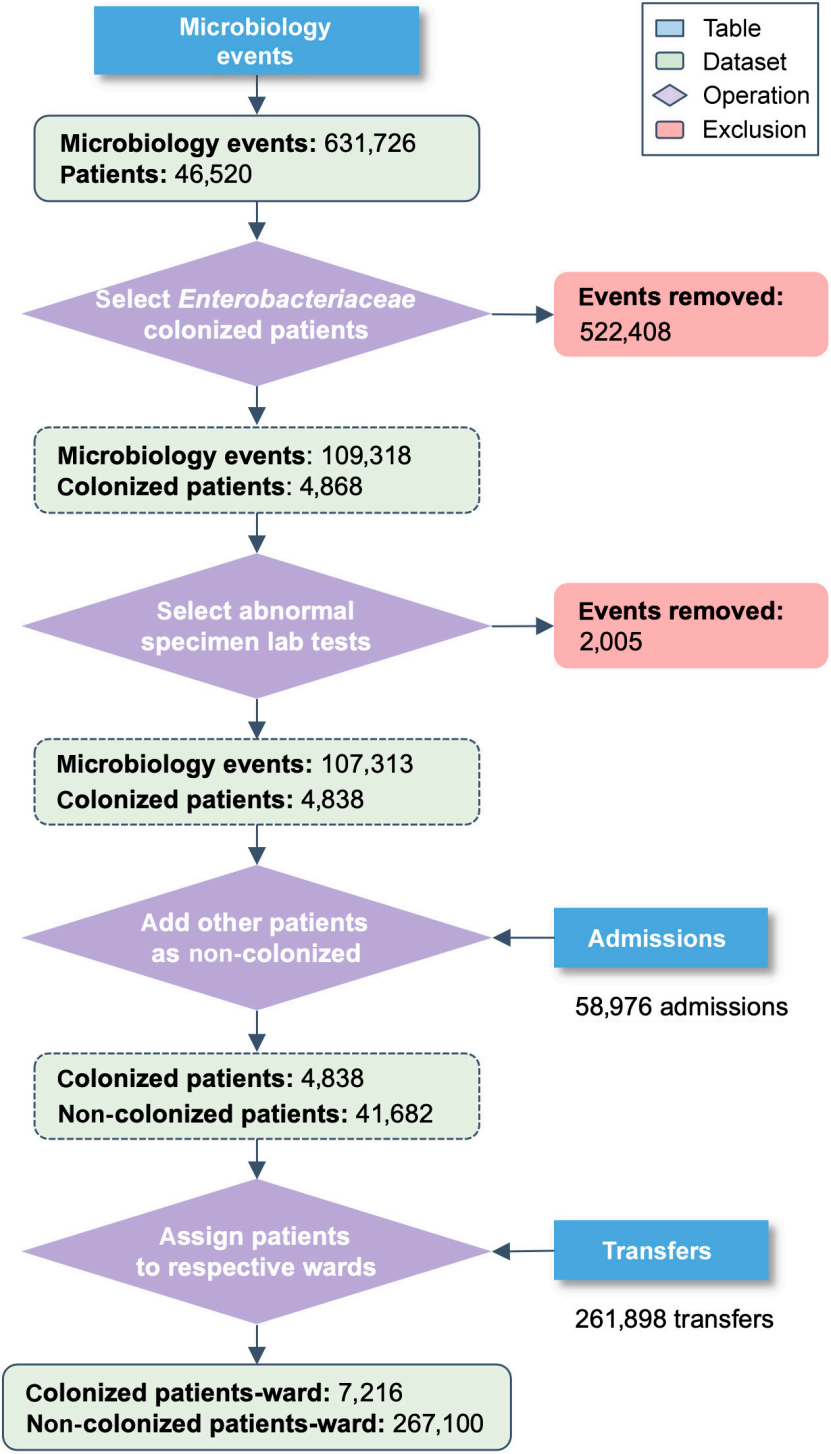
Cohort selection criteria. Starting from the Microbiology Events table of MIMIC-III, lab results were filtered for the presence Enterobacteriaceae in unusual body parts to define colonized patients. Admissions and Transfers tables were used to identify the remaining patients and to label all patients.

The training and evaluation dataset used in this study was created using the cohort selection criteria described in Fig. 2. The Microbiology Events table from MIMIC-III was used to detect positively colonized patients. The table contains bacterial identification and antimicrobial testing results and consists of 631,726 events related to 46,520 patients. A list of Enterobacteriaceae species was selected using the National Center for Biotechnology Information terminology [36] and used to select the microbiology events of patients colonized by Enterobacteriaceae. This first step resulted in 109,318 events related to 4,868 colonized patients. Then, a list of abnormal specimens (or uncommon body parts) where these species were found was identified by 2 clinical microbiologist experts and categorized into 6 specimen categories: blood, gastric-related, respiratory, skin, tissue, and urine. This list defined the set of positive events that were relevant to our study, i.e., the presence of Enterobacteriaceae in abnormal body parts, resulting in 107,313 microbiology events and 4,838 colonized patients. The Admissions table details hospitalizations of every patient in the database, and was used to define the remaining non-colonized patients. Among all admitted patients, the ones that were not found in the filtered Microbiology Events table, in addition to those with Enterobacteriaceae in regular specimens (i.e., stool samples), were considered non-colonized. Lastly, the table Transfers*,* which contains patient location information and their transfers between wards, was used to assign patients to wards. The final dataset contained 46,520 unique patients from 58,976 admissions, and a total of 274,316 patient–ward instances. If, during a stay in a ward, there was no positive abnormal Enterobacteriaceae culture for a patient, the patient–ward instance was labeled as non-colonized; otherwise, it was labeled as colonized. This resulted in 7,216 positive Enterobacteriaceae colonizations (2.6%) and 267,100 negative specimens (97.4%). The dataset was randomly divided into train (60%), dev (20%), and test (20%) splits to train model parameters, optimize model hyper parameters, and evaluate model performance, respectively. Each split contained 2.5% to 3% colonized patients.

### Feature selection and data pre-processing

The feature selection process was performed iteratively. The MIMIC-III dataset was first analyzed to pre-identify the set of features we considered relevant to the colonization risk prediction problem. Then, based on model performance computed with the dev set, less important features, such as the time of death and the discharge status of the patient, were eliminated. The selection converged to 2 types of features: (a) spatiotemporal features (current and previous ward, current and previous care unit, length of stay in each ward and in the hospital) and (b) patient features (gender and diagnosis at admission). To complement this set, we computed 3 new features from the data: the number of colonized patients, the total number of patients per ward, and the colonization pressure [37]. The latter was calculated as the ratio of colonized and the total number of patients in a ward per day. Finally, the features were normalized using the robust scaler method of scikit-learn [38], version 1.1.2. Table 1 shows the statistics of the resulting dataset.

**Table 1.** Statistics of the cohort used for model training and evaluation

Model	Data balance	Setting	Links	Accuracy (%)	Sensitivity (%)	Specificity (%)	AUROC (%) (95% CI)
Logistic regr.	Non	-	-	82.37	74.74	82.58	87.92 (87.17–88.70)
Logistic regr.	Non	Inductive	Out-ward	89.19	63.43	89.89	88.11 (87.41–88.96)
Logistic regr.	Non	Transductive	In-ward	80.77	78.42	80.84	88.59 (87.93–89.32)
k-NN	Under	-	-	80.15	82.10	80.10	90.14 (89.35–90.83)
k-NN	Under	Inductive	In-ward	86.23	67.59	86.73	87.66 (86.93–88.52)
k-NN	Under	Transductive	In-ward	72.46	81.61	72.22	86.43 (85.57–87.22)
Random forest	Non	-	-	97.68	14.92	99.91	90.97 (90.27–91.59)
Random forest	Non	Inductive	Out-ward	84.57	70.51	84.94	86.99 (86.22–87.79)
Random forest	Over	Transductive	In-ward	97.70	14.43	99.95	89.78 (89.32–90.44)
CatBoost	Non	-	-	97.68	16.79	99.86	90.55 (89.94–91.27)
CatBoost	Non	Inductive	Out-ward	74.89	84.52	74.63	88.64 (88.04–89.46)
CatBoost	Over	Transductive	In-ward	97.83	23.39	99.84	90.94 (90.24–91.69)
GNN	Non	-	No	82.47	79.94	82.53	88.66 (87.98–89.43)
GNN	Non	Inductive	In-ward	82.74	83.00	82.73	91.23 (90.61–91.85)
GNN	Non	Transductive	In-ward	96.18	80.57	96.60	96.13 (95.63–96.60)

### Colonization network model

**Fig. 3.**
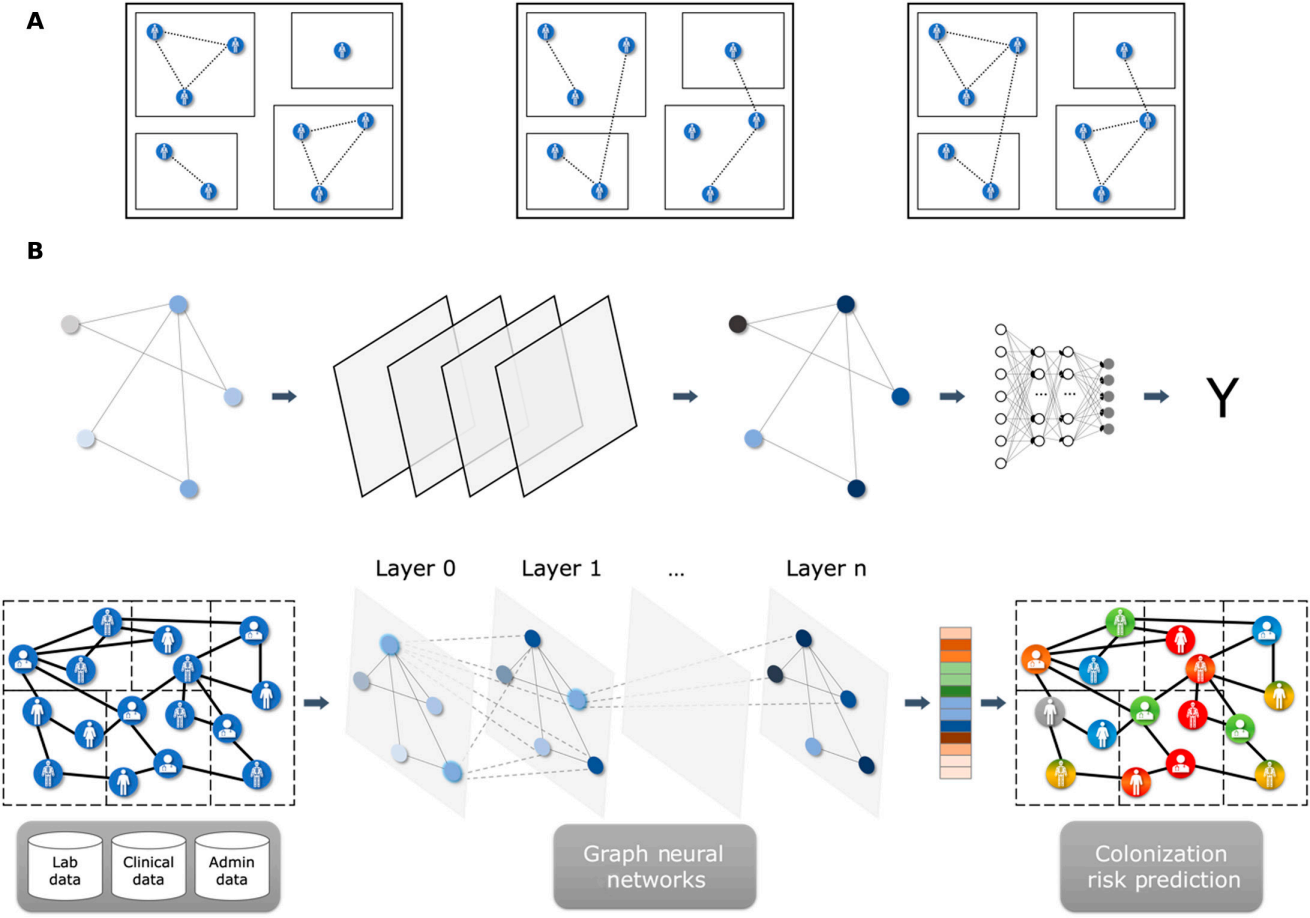
(A) Colonization models. We constructed 3 different graphs, in which links were created between patients only if they were in the same ward (left), only if they were visited by the same healthcare worker (center), or both (right). (B) Graph-based machine learning pipeline for colonization risk prediction.

We propose a homogeneous graph to model interactions between patients and healthcare workers. A graph can be defined as *G* = (*V*, *E*), where *V* = *v*_1_, …, *v*_∣*V*∣_ denotes a set of nodes and *E* denotes a set of edges connecting pairs of nodes *v_i_*, *v_j_* ∈ *V*. In our case, a node represents a patient in a ward and edges represent potential connections between patients, either via contacts with the same healthcare worker or via a common location within the hospital. As shown in Fig. 3A, we considered 3 network configurations: (a) in-ward links (left), where 2 patients are linked only if they stay in the same ward at the same time; (b) out-ward links (middle), where 2 patients are connected only if they are visited by the same healthcare worker on the same day; and (c) all links (right), where both ward and healthcare worker links are considered. Nodes represent a patient in a ward and their features are created using the selected set of features described in the previous subsection. For each patient transfer, a new node is added to the graph and edges are set following the network configuration (i.e., in-ward, out-ward, or all links).

### GNN architecture

GNN [32,39–41] is an elegant deep learning architecture for modeling graph-like data structures and learning topological features, i.e., in our case, properties of the transmission network. GNNs can learn complex relationships and interdependencies in graph-like data via optimizable transformations on attributes (nodes, edges, etc.) that preserve graph symmetries (i.e., permutation invariance/equivariance). Hence, in theory, GNNs can make more informed predictions about entities in a network and their interactions, as compared to models that consider entities in isolation. One distinct advantage of graph-based techniques is their ability to perform inductive and transductive learning. In inductive learning, models learn to generalize to unseen nodes or graphs, whereas the transductive approach includes test nodes (not including true labels) in the graph during training. The disadvantage of the transductive setting is that the model must be retrained for each new instance.

To solve graph representation learning tasks, different GNN network architectures and algorithms have been proposed, such as graph convolutional network (GCN) [32], graph attention networks (GAT) [42], and GraphSAGE [43]. These approaches use various graph feature aggregation and data sampling strategies to learn dense representations of graph components (i.e., nodes and edges), often called embeddings, that can be later used in downstream prediction tasks, such as node classification. Each layer of a GNN aggregates a subset of the nodes of the previous layer to generate an updated feature vector, for each node. GCN layers average the features of neighboring nodes in the previous layer, akin to convolutional neural networks. They can efficiently capture local graph structures and scale to large graphs. GAT layers extend GCNs by adding an attention mechanism. Instead of treating all neighbors equally as in GCN layers, GAT dynamically computes the weights of the node aggregation process, based on node features. They are hence able to capture the local structure of the graph in a more nuanced way. GraphSAGE layers generate embeddings by sampling and aggregating features from a node's local neighborhood. The advantage of GraphSAGE is that it can learn from very large graphs and generate embeddings for unseen nodes, allowing the network to generalize to larger datasets.

In our experiments, we let the type (GCN, GAT, and GraphSAGE), number (from 2 to 5), and hidden dimension (16, 32, 64, 128, and 256) of the GNN layers as hyper-parameters. A high-level view of the graph-based prediction pipeline is shown in Fig. 3. Using laboratory, clinical, and administrative data, patient features at the ward level (since a node represents a patient in a ward) were extracted and modeled in different network colonization models (Fig. 3A). The colonization graph was fed to the GNN, which performed the node classification task. Each GNN layer was followed by a rectified linear unit (ReLU) operation and a dropout layer, whose probability was used as a hyper-parameter (0.1, 0.2, 0.3, 0.4, and 0.5). Finally, each node’s feature vector of the last GNN layer was sent to a sigmoid layer for node classification (Fig. 3B). To account for data imbalance, we used a weighted focal loss[44]. The weight of each sample was set to 1 for non-colonized samples and to a hyper-parameter (from 10 to 1,000) for colonized samples. Moreover, we tried different data balance scenarios (original data, over-sampling of the minority class, and under-sampling of the majority class). We trained all models for 500 epochs, using the AdamW [45] optimizer with a learning rate that was set as a hyper-parameter (from 0.001 to 1.0). We had different conditions defined by the type of network we used as data (all links, ward links only, and caregiver links only), the type of settings in which the network was trained and evaluated (transductive or inductive), and the type of data balance scenario. For each of these conditions, all hyper-parameters of the GNN model were optimized using Optuna [46], sampling hyper-parameters for 100 trials with the TPESampler [47] algorithm to maximize AUROC, computed with the dev set. The best set of hyper-parameters identified for each condition is available at https://github.com/ds4dh/hai_project/tree/master/models/gnn.

### Statistical analysis

To evaluate the performance of the colonization risk prediction models, we computed metrics typically used in medical contexts, where it is crucial to understand both the ability to correctly identify positive and negative cases: sensitivity, specificity, and AUROC. The GNN models were compared to classic machine learning baselines: k-nearest neighbors (k-NN) [48], logistic regression [49], random forest [50], and CatBoost [51]. As for GNN simulations, the hyper-parameters of the baseline models were optimized using Optuna [46], with 100 trials for each model, sampling hyper-parameters with the TPESampler [47] algorithm. The best set of hyper-parameters identified for each baseline model was made available at https://github.com/ds4dh/hai_project/tree/master/models/controls. We also provide simulations that include edge information as node2vec [34] feature vectors, concatenated to the original input features of the baseline models. For any GNN or baseline model, confidence intervals were computed for AUROC using bootstrapping (number of bootstraps = 100, alpha level = 0.05). Shapley values were used to measure the importance of each feature to the model’s predictions.

## Results

### Performance of baseline and GNN models

Table 2 shows results obtained with the different colonization risk prediction models. For each model, we report the data balance scenario (no balance, over-sampling, and under-sampling) that led to the best performance. Except for k-NN, over- or under-sampling did not improve performance for any model. In addition to the individual classic and graph-based models, we created an ensemble model, which combines the results of classic machine learning models and GNN. We tried different model combinations and the one that led to the best performance was an ensemble of CatBoost, random forest, and GNN trained in an inductive setting, using ward links. We did not use GNN trained in a transductive setting, since it is not evaluated in the same way as other models. The predicted class probabilities of each selected model were averaged to generate a prediction. We tried different voting strategies (unanimity, majority), which were inferior to the averaging strategy.

**Table 2.** Performance of the different colonization prediction models. Ensemble model includes predictions from inductive-GNN, random forest, and CatBoost

Model	Data balance	Setting	Links	Accuracy (%)	Sensitivity (%)	Specificity (%)	AUROC (%) (95% CI)
Logistic regr.	Non	-	-	82.37	74.74	82.58	87.92 (87.17–88.70)
k-NN	Under	-	-	80.15	82.10	80.10	90.14 (89.35–90.83)
Random forest	Non	-	-	97.68	14.92	99.91	90.97 (90.27–91.59)
CatBoost	Non	-	-	97.68	16.79	99.86	90.55 (89.94–91.27)
GNN	Non	Inductive	All	84.23	78.21	84.39	89.67 (88.87–90.35)
GNN	Non	Inductive	In-ward	82.74	83.00	82.73	91.23 (90.61–91.85)
GNN	Non	Inductive	Out-ward	84.89	71.55	85.25	87.90 (87.14–88.67)
GNN	Non	Transductive	All	92.36	80.50	92.68	94.07 (93.59–94.60)
GNN	Non	Transductive	In-ward	96.18	80.57	96.60	96.13 (95.63–96.60)
GNN	Non	Transductive	Out-ward	83.55	79.81	83.65	89.36 (88.62–89.98)
Ensemble	Non	Inductive	In-ward	97.43	31.16	99.22	92.17 (91.68–92.72)

Graph-based models tend to outperform the baseline models. Particularly, the GNN trained in a transductive setting achieves the best performance overall (96.13% AUROC with in-ward links). Importantly, the GNN trained in an inductive setting using in-ward links also outperforms baseline models (91.23% AUROC). Comparing different types of links for the GNN models, in-ward links produce the best performance, both in a transductive and in an inductive setting. These results suggest that network features enhance the predictive power of machine learning models for colonization risk prediction, and that transmission patterns within the same ward are more useful features. Note that, for all GNN conditions reported in Table 2, the type of GNN layer that was selected by the hyper-optimization process was always GraphSage. Results using different layer types are shown in Table S1. Finally, the ensemble model—combining CatBoost, random forest, and GNN trained in an inductive setting and using in-ward links—improves performance upon individual models (92.17% AUROC).

For the metrics with a decision threshold, we report that, for each model, the performance obtained with a threshold of 0.5. k-NN achieves the highest accuracy (97.68%) and specificity (99.91%). The GNN model trained in an inductive setting with in-ward links obtains the best sensitivity (83.00%), at the cost of lower accuracy (82.74%) and specificity (82.73%). In comparison, baseline models either achieve inferior performance (logistic regression and k-NN), or a more unbalanced trade-off between sensitivity and specificity (random forest and CatBoost). Finally, GNN trained in a transductive setting with in-ward links yields not only high specificity (80.57%), but also high accuracy (96.18%) and sensitivity (96.60%). Overall, GNN models achieve a more balanced predictive performance between colonized and non-colonized patients, which, together with a high enough accuracy, may foster better practical applications (at the expense of a reduced assessment set).

### Stratified performance analysis for the GNN model

**Fig. 4.**
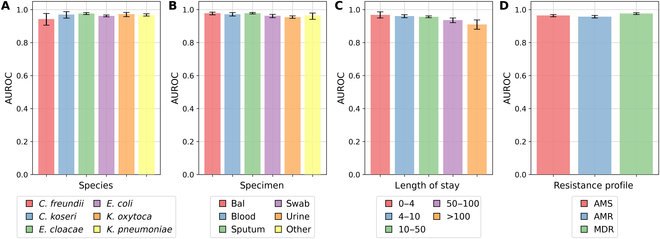
Performance results per (A) species, (B) specimen type, (C) length of stay (expressed in days), and (D) resistance profile. Bal, bronchoalveolar lavage; AMS, antimicrobial susceptible; AMR, antimicrobial resistant; MDR, multidrug resistant.

Figure 4 shows AUROC for the best individual model—GNN trained in a transductive setting and using in-ward links only—where the test dataset was stratified by species, specimen type, length of stay and resistance profile. The results show that the model provides consistent performance across different bacteria species, with 96.56% overall AUROC for species that have at least 10 examples in the training dataset. The best performance is observed for *E. cloacae* (97.64%) (183 examples in the testing set) while the worse is for *Citrobacter freundii* (94.33%) (41 examples in the testing set). Similarly, consistent performance is observed across specimens, with AUROC varying from 95.52% for urine culture to 97.87% for bronchoalveolar lavage. The results of Fig. 4C show a decreasing trend in performance as patients stay longer in the hospital, with AUROC as high as 96.88% for patients that stay 4 days or less and as low as 91.02% for patients that stay more than 100 days. Lastly, the model achieves similar predictive performance for different resistance profiles with the lowest AUROC at 95.73% for AMR Enterobacteriaceae and the highest score at 97.62% for MDR Enterobacteriaceae.

### Predictive performance for AMS, AMR, and MDR resistance profiles

**Fig. 5.**
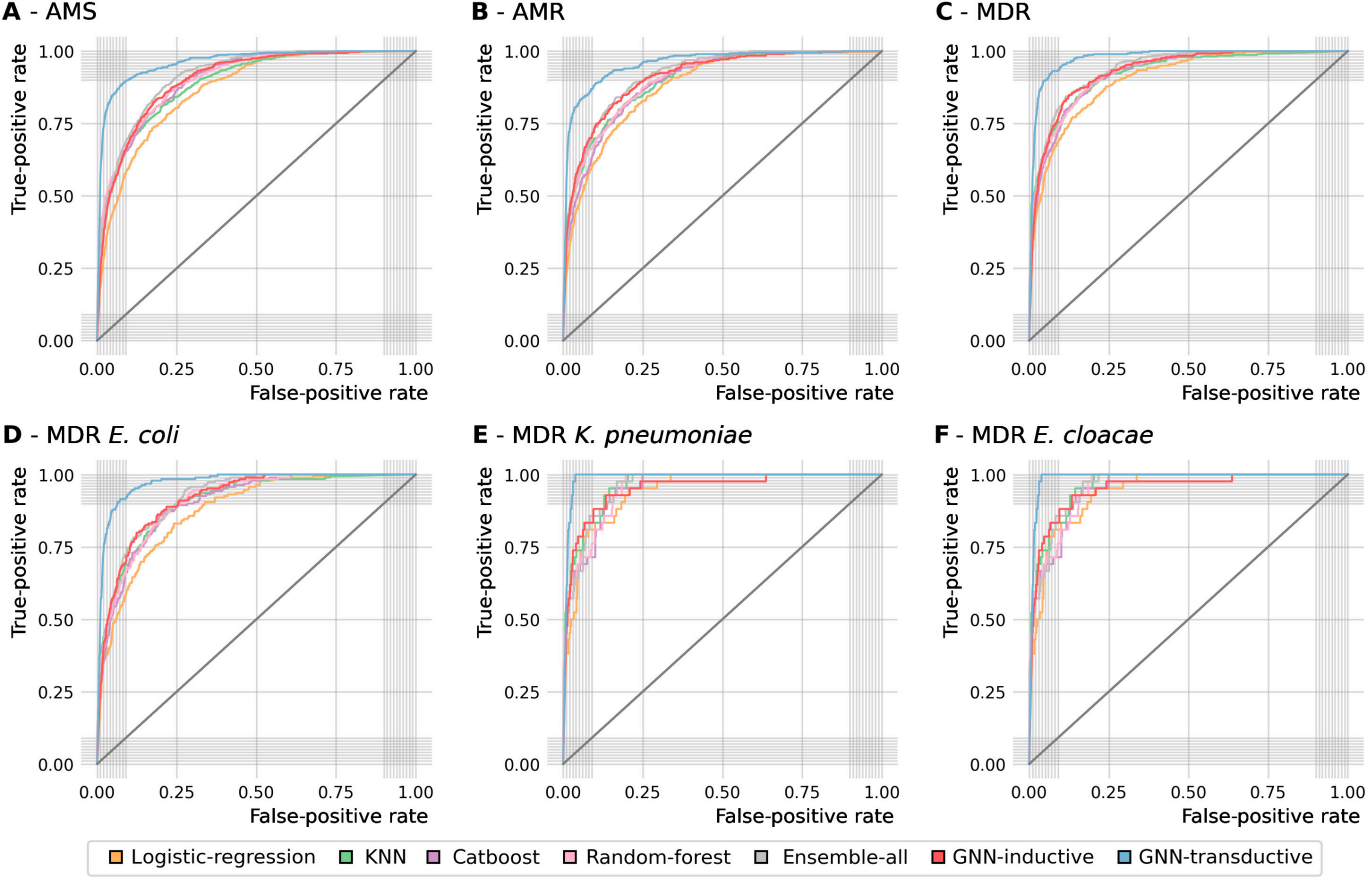
Model performance for (A) antimicrobial susceptible (AMS), (B) antimicrobial resistant (AMR), and (C) multidrug-resistant (MDR) Enterobacteriaceae, and for representative MDR bacteria: (D) *E. coli*, (E) *K. pneumoniae*, and (F) *E. cloacae*.

The predictive performance for AMS, AMR, and MDR resistance profiles and for the 3 most frequent MDR Enterobacteriaceae species is shown in Fig. 5. Like the general case (see Table 2), the GNN model trained in a transductive setting is the best model overall (blue curves) and the logistic regression model is the worst model overall (orange curves). The ensemble model and the GNN model trained in an inductive setting come second (gray and red curves). The ensemble model slightly outperforms the inductive GNN, especially for AMS Enterobacteriaceae.

### Baseline models with node2vec embeddings

As the baseline models in Table 2 do not have access to edge information, we also controlled that using GNN layers is the best way to exploit the topological features of the patient network. For this reason, we included baseline model simulations in which edge information is concatenated to the original input features. We used the node2vec [34] algorithm to embed network information into feature vectors. The node2vec algorithm randomly walks on the network to generate sequences that represent the neighborhood of each node. A skip-gram [52] model is trained with these node sequences and learns to produce feature vectors that preserve both the local and global structure of the network. The parameters of node2vec were the following: number of features = 128, skip-gram window size = 5, maximum random walk length = 32, number of random walks per root node = 10, p = 0.5, and q = 2.0. Table 3 shows the performance of baseline models trained with this updated set of features. We report, for each model, the data balance scenario (no balance, over-sampling, and under-sampling) and link condition (in-ward, out-ward, and all) that led to the best performance.

**Table 3.** Performance of the baseline models that use edge features as node2vec embeddings, concatenated to the original set of features, as compared to the best GNN models

Model	Data balance	Setting	Links	Accuracy (%)	Sensitivity (%)	Specificity (%)	AUROC (%) (95% CI)
Logistic regr.	Non	-	-	82.37	74.74	82.58	87.92 (87.17–88.70)
Logistic regr.	Non	Inductive	Out-ward	89.19	63.43	89.89	88.11 (87.41–88.96)
Logistic regr.	Non	Transductive	In-ward	80.77	78.42	80.84	88.59 (87.93–89.32)
k-NN	Under	-	-	80.15	82.10	80.10	90.14 (89.35–90.83)
k-NN	Under	Inductive	In-ward	86.23	67.59	86.73	87.66 (86.93–88.52)
k-NN	Under	Transductive	In-ward	72.46	81.61	72.22	86.43 (85.57–87.22)
Random forest	Non	-	-	97.68	14.92	99.91	90.97 (90.27–91.59)
Random forest	Non	Inductive	Out-ward	84.57	70.51	84.94	86.99 (86.22–87.79)
Random forest	Over	Transductive	In-ward	97.70	14.43	99.95	89.78 (89.32–90.44)
CatBoost	Non	-	-	97.68	16.79	99.86	90.55 (89.94–91.27)
CatBoost	Non	Inductive	Out-ward	74.89	84.52	74.63	88.64 (88.04–89.46)
CatBoost	Over	Transductive	In-ward	97.83	23.39	99.84	90.94 (90.24–91.69)
GNN	Non	-	No	82.47	79.94	82.53	88.66 (87.98–89.43)
GNN	Non	Inductive	In-ward	82.74	83.00	82.73	91.23 (90.61–91.85)
GNN	Non	Transductive	In-ward	96.18	80.57	96.60	96.13 (95.63–96.60)

Some baseline models achieve slightly higher AUROC when edge information is added to the original features (logistic regression and CatBoost). Still, transductive and inductive GNN models both outperform any combination of baseline model and added edge features, in terms of AUROC. In terms of accuracy, sensitivity, and specificity, some combinations improve the performance of baseline models. However, as for Table 2, these combinations either achieve inferior performance, or a more unbalanced trade-off between sensitivity and specificity. The best baseline combination is arguably CatBoost with inductive out-ward links, which marginally outperforms inductive GNN in terms of sensitivity (84.52%) but whose specificity drops significantly (74.63%). Additionally, AUROC for this combination (88.64%) is notably lower than that of GNN models. Finally, we include a simulation in which GNN have no edges. GNN show a more significant AUROC performance boost from added edges compared to baseline models, indicating they handle network information better than merely adding node2vec [34] features.

### Feature impact on model predictions

**Fig. 6.**
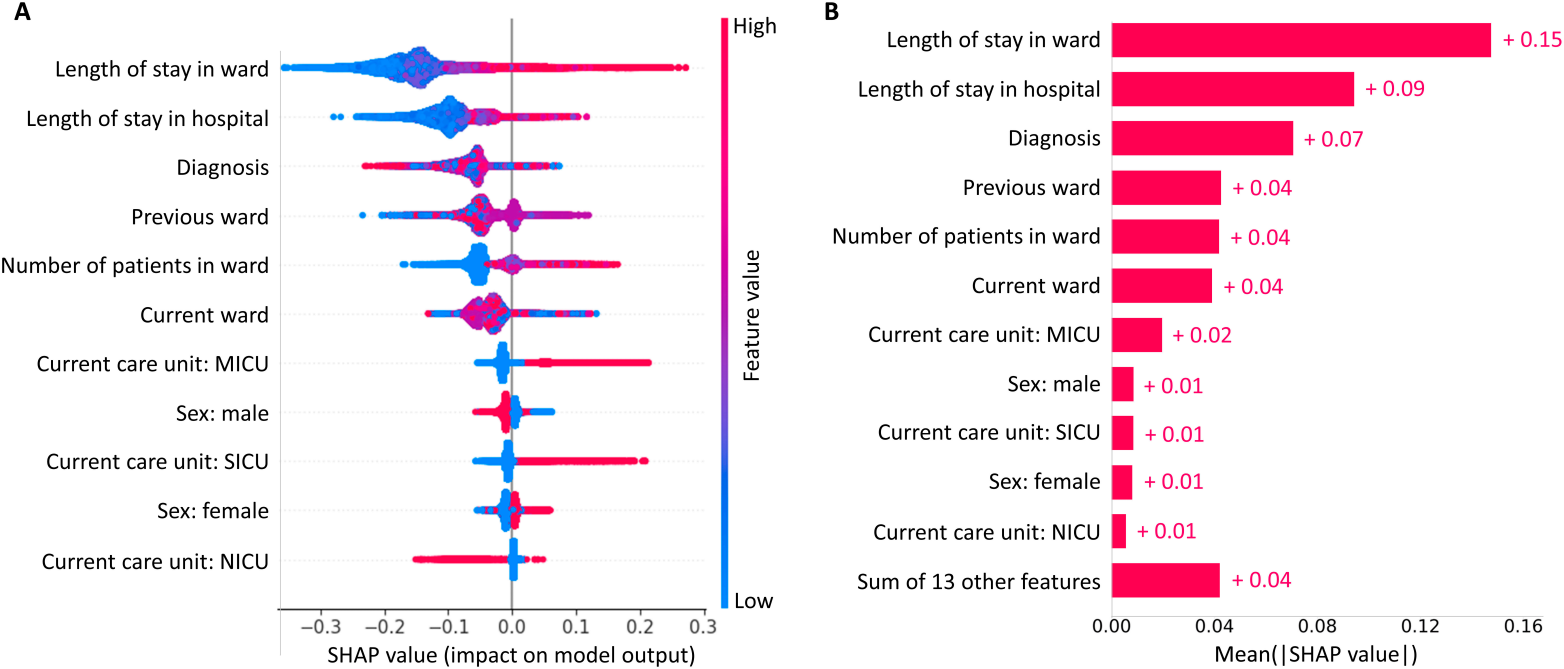
Feature contribution to colonization risk prediction. (A) Shapley values for the top 11 features, sorted by their impact on model predictions. (B) Mean absolute value of every feature presented in (A).

To explain the importance and impact of the features used in our colonization risk prediction models, we calculated Shapley values using the SHAP method [53]. For simplicity, we used the results of the random forest model, as the baseline model with the highest AUROC. Figure 6A shows the importance of the top 11 features sorted by their predictive impact. Figure 6B shows the mean absolute value of every feature presented in Fig. 6A, computed over all data samples. As expected, length of stay in the ward and in the hospital has high impact on model predictions. Indeed, the longer the stay in a ward or hospital, the more likely it is for a patient to be classified by the model as colonized. The number of patients in a ward also has a large impact on model predictions. The higher the number of patients in the ward, the more probable the model output to be positive (colonized). Despite its lower impact, female gender influenced the model output in the positive (colonized) direction compared to male, which has the opposite effect. This could be explained by the fact that the most prevalent bacteria in the dataset were *E. coli* and that urinary tract infections are more common among women than men [54]. Similarly, the neonatal intensive care unit (NICU) was less important to the model decisions than the medical intensive care unit (MICU) and surgical intensive care unit (SICU). A patient in SICU and MICU will more likely drive the model toward a positive output (colonized), while a patient in NICU will more likely drive the model toward a negative output (non-colonized). These findings are aligned with previous risk factor analysis studies for nosocomial infections in adult intensive care units [55]. Lastly, it is noteworthy that many of the key features identified by the Shapley analysis pertain to ward-related information. This aligns with the observation that in-ward links yield the best performance in GNNs.

### Colonization path analysis

**Fig. 7.**
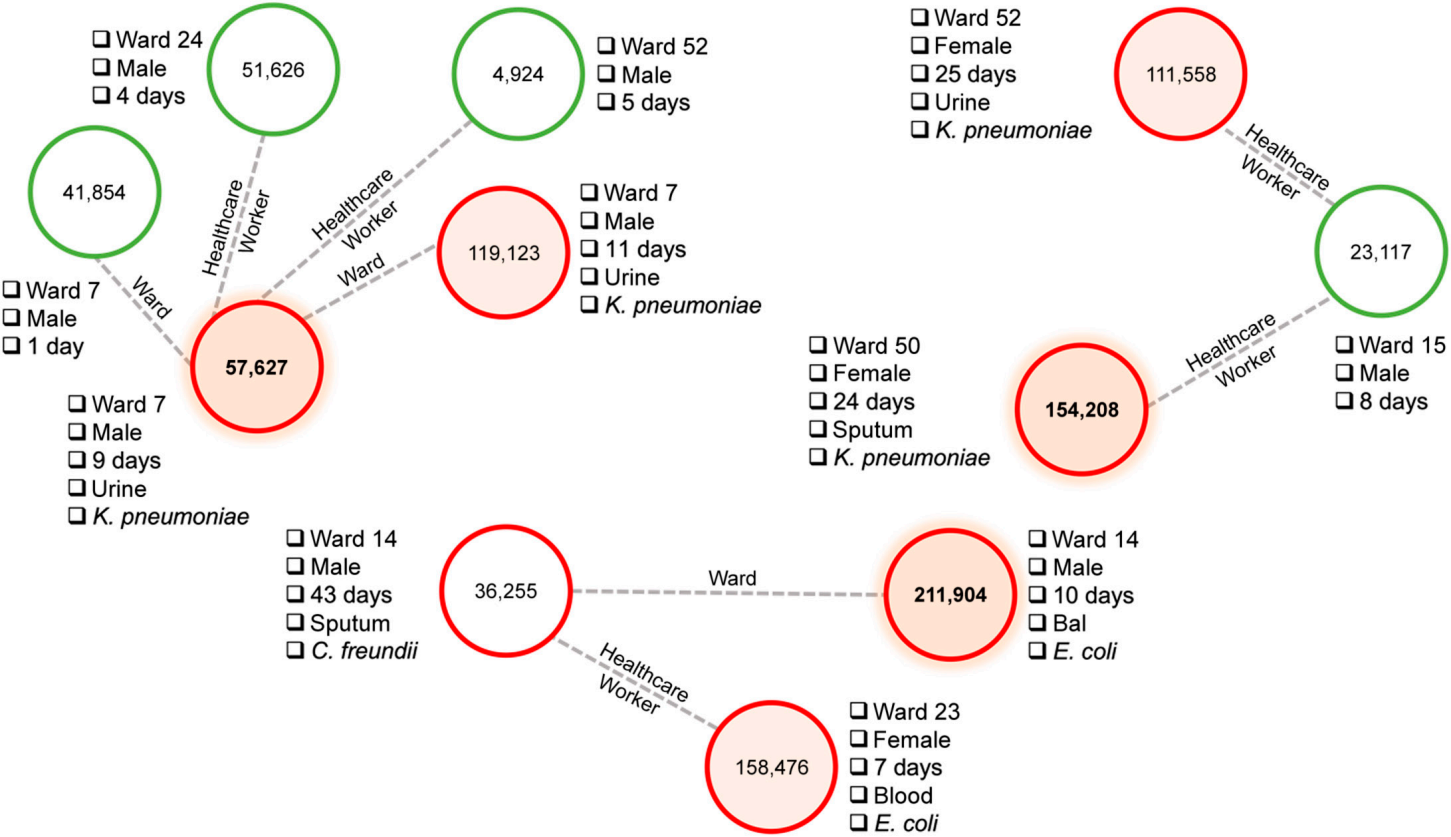
Bacteria transmission scenarios via graph paths. Green nodes: non-colonized patients; red nodes: colonized patients.

A major advantage of using graph models and GNNs to predict colonization risks is that they naturally provide possible transmission paths via graph edges. In Fig. 7, we show 3 examples of patients that were classified correctly as colonized by the transductive GNN model: nodes 57,627 (top left), 154,208 (top right), and 211,904 (bottom). Nodes in green represent non-colonized patients and nodes in red represent positive culture for Enterobacteriaceae. Filled colors represent colonized patients. In the scenario of Fig. 7, top left, patient 57,627 (focus patient hereafter), who was colonized by *K. pneumoniae*, stayed in the hospital for 9 days and was directly linked to 4 patients: 2 in the same room (1 non-colonized and 1 colonized) and 2 in different rooms (both non-colonized). Similar to the focus patient, patient 119,123 was colonized by *K. pneumoniae* and had the longest hospital stay in this subnetwork (11 days). Thus, if both bacterial strains were genetically identical, a possible transmission route could have been from patient 119,123 to the focus patient or vice versa, or from a common source within the ward environment or utility. In Fig. 7, top right, patient 154,208 (focus patient hereafter) stayed for 24 days in the hospital and had an immediate link to patient 23,117 (non-colonized) from a different ward via a healthcare worker, and a second-degree connection to patient 111,558 (colonized) from another ward. The latter patient and the focus patient were both colonized by *K. pneumoniae*, like in the previous scenario. Hence, path 111,558–23,117–154,208 could be one of the possible transmission routes within the hospital. For the third scenario, Fig. 7, bottom, patient 211,904 (focus patient hereafter), male, stayed for 10 days and had a direct connection to patient 36,255 via the same ward, both colonized, but by different bacteria. Moreover, these patients had a second-degree connection to patient 158,476, female, via a healthcare worker link, who was colonized by *E. coli*, as the focus patient. Since patient 158,476 was hospitalized for 7 days, she may have been colonized by the same strain of the focus patient (or vice versa), who may have been previously colonized. Thus, the undirected path 211,904–36,255–158,476 could be a possible transmission route. Nevertheless, exact identification of transmission routes for such scenarios would require detailed phylogenetic analysis of bacterial samples [56], not available in the MIMIC-III database.

## Discussion

This study describes a machine learning model based on GNNs to predict patients at risk of colonization by AMR and MDR Enterobacteriaceae. We model the data as a graph to represent possible connections and interactions between patients and healthcare workers inside the healthcare facility. Different graph topologies were proposed based on geographic location and interaction with healthcare workers. We considered spatiotemporal features, such as length of stay and ward movement, in addition to clinical and laboratory information, to encode patients via node features in different graph topologies. Performance analyses showed that GNN models provide robust predictive performance, often above AUROC of 90%, outperforming the classic machine learning baselines used in our experiments. These results demonstrate the importance of incorporating topological features to learn patterns of patient profiles that are more likely to be colonized by MDR Enterobacteriaceae.

Other recent studies investigated the use of machine learning to predict colonization risk of AMR species from Enterobacteriaceae [28,29], Enterococcaceae [27], and Staphylococcaceae [19] families, achieving robust predictive performance with an AUROC between 88% and 89%. Our study is the first to consider the colonization risk for AMR and MDR Enterobacteriaceae family, responsible for the highest incidence of nosocomial infections and HAI-related mortality [57], using a transmission network approach and spatiotemporal information. Moreover, in contrast to previous studies, which were based on ensemble of tree methods such as random forest, our proposed methodology used a deep learning approach and showed superior predictive power for the colonization prediction problem of Enterobacteriaceae. Another advantage of graph-based modeling, as opposed to tabular data used in previous studies, is that possible transmission routes can be inherently extracted from the model, opening an avenue for data-driven transmission route hypothesis generation.

Following IPC guidelines, when an AMR Enterobacteriaceae outbreak occurs in a hospital or in a long-term care facility, colonized patients are initially isolated. Then, the contact group, i.e., patients potentially colonized by the outbreak strain, is identified to determine the magnitude of the outbreak and, if required, additional IPC measures are applied [58]. Using administrative information from the EHR system, contact tracing information can be obtained and used to determine other patients potentially at risk, which will ultimately go through a screening process to duly confirm colonization. This process is reactive and can be uncomfortable for patients, as well as very costly and time consuming, thus preventing corrective actions from being taken in due time [59]. The predictive model proposed in this study could help improve IPC measures against Enterobacteriaceae, and other pathogens, in several ways. First, it could help to estimate the contact group with high accuracy, which, in turn, could lead to more effective measures to curb transmission and infection. Second, possible transmission paths could be automatically derived from the graph model, providing hypotheses for transmission routes. Lastly, and more importantly, if deployed in a surveillance mode, it could support early identification of potential patients at risk of AMR and MDR colonization and enable outbreak forewarning, which can deliver an even higher positive impact on life-saving and financial costs.

Explainable artificial intelligence methods, such as the Shapley values used to analyze our results, can provide an effective approach to interpret the model decisions and support the identification of risk factors associated with colonization risks. Among the features having the highest impact on model predictions, features such as length of stay, previous ward, and gender have also been identified as relevant by previous epidemiological studies that investigated risk factors for HAI colonization and infection. For example, Patel et al. [60] showed that carbapenem-resistant *K. pneumoniae* infection was independently associated with longer length of stay before infection. McHaney-Lindstrom et al. [61] showed that unit transfer increases the odds of contracting an infection by 7%. For the case of gender, the model not only identified this feature as a risk factor but also showed that being female is associated with higher risk of Enterobacteriaceae colonization. This result was found in previous risk analysis studies, which identified higher incidence rates of *E. coli* in women as compared to men [62].

Applying machine learning algorithms to solve the task of colonization risk prediction is challenging due to the imbalanced nature of the data. Machine learning models are often biased toward the majority class (i.e., non-colonized in our case), and in the worst-case scenario, they will ignore the minority group entirely. Consequently, relying solely on accuracy can be misleading when evaluating model performance. A model that completely fails to predict the minority class (in this case, the colonized group) could still obtain high accuracy. Yet, given the consequences of false positives and negatives in a real-case scenario, such a model would not be useful. False positives may lead to unnecessary isolation of patients and exposure to unwanted risks and side effects, whereas false negatives could result in the spread of a pathogen, delayed treatments, and worsened patient outcomes. To provide a more comprehensive view of our results, we reported sensitivity and specificity, which help understand both the ability of a model to correctly identify positive (colonized) and negative (non-colonized) cases, and AUROC, which offers a more holistic view on model outcomes. GNNs outperform all baseline models in terms of AUROC. Moreover, GNNs trained in an inductive setting tend to yield more balanced outcomes compared to classic models by achieving a higher sensitivity at the cost of a lower specificity. In contrast, GNN trained in a transductive setting enhances sensitivity compared to baseline models while maintaining high accuracy and specificity, at the cost of a more complicated training process.

Our study has several limitations, in terms of both data and modeling. First, the model might not be able to generalize to other hospitals as it was only evaluated in a single hospital unit dataset. Indeed, it is known that the epidemiology of HAI varies within different units and geographies [63]. Investigations of generalization performance for this type of models will warrant specific future research. Second, while we avoided using predictors that might overlap with the dependent variable, such as antimicrobial consumption (e.g., trimethoprim-sulfamethoxazole antimicrobial medication could be a predictor for *E. coli*[64]), other predictors, such as diagnosis at admission, could still have caused prediction bias. Nevertheless, given the distribution of diagnoses in the dataset, we expect that this bias is limited, if any. Third, our graph topology does not include environmental transmission, while it is known that indirect transmission via the environment is an important part of HAI routes [65]. Due to the lack of fine-grained contact and sampling data in the MIMIC-III dataset, environment-related transmission pathways were ignored in our models as this scenario could not be realistically captured. Understanding the impact of environmental transmission on model performance could be another research direction. Lastly, due to the anonymization strategy of MIMIC-III and, more specifically, to the randomized time shifts, the data used in our experiments could be better regarded as synthetic data (generated from real data) rather than as real hospital data [66,67].

To conclude, we show that encoding topological information about patient interactions using GNNs can improve the predictive performance of AMR and MDR Enterobacteriaceae colonization models and support the identification of patients potentially at risk of colonization or infection. Hence, these models could be used to enhance IPC programs and reduce HAI burden. Given the data-driven approach of our method, we expect that it could be expanded to other pathogens with similar transmission dynamics and to other healthcare settings.

## Ethical Approval

This study used the MIMIC-III database, which is publicly available and deidentified, thus exempt from institutional review board approval. Researchers involved in this study completed the required training to access and work with the MIMIC-III data.

## Data Availability

The MIMIC-III database is freely and publicly available through PhysioNet. The code used to produce the results presented in this study is available at https://github.com/ds4dh/hai_project.

## References

[1] Allegranzi B, Nejad SB, Combescure C, Graafmans W, Attar H, Donaldson L, Pittet D. Burden of endemic health-care-associated infection in developing countries: Systematic review and meta-analysis. Lancet. 2011;377(9761):228–241. 21146207 10.1016/S0140-6736(10)61458-4

[2] World Health Organization. *Charter: Health worker safety: A priority for patient safety*. Geneva (Switzerland): World Health Organization; 2020.

[3] World Health Organization. *Report on the burden of endemic health care-associated infection worldwide*. Geneva (Switzerland): World Health Organization; 2011.

[4] Klevens RM, Edwards JR, Richards CL Jr, Horan TC, Gaynes RP, Pollock DA, Cardo DM. Estimating health care-associated infections and deaths in US hospitals, 2002. Public Health Rep. 2007;122(2):160–166. 17357358 10.1177/003335490712200205 PMC1820440

[5] Patient Carelink. Healthcare-acquired infections (HAIs). 2022. Available at http://patientcarelink.org/improving-patient-care/healthcare-acquired-infections-hais/ [accessed October 10, 2022].

[6] Tzouvelekis LS, Markogiannakis A, Piperaki E, Souli M, Daikos GL. Treating infections caused by Carbapenemase-producing Enterobacteriaceae. Clin Microbiol Infect. 2014;20(9):862–872. 24890393 10.1111/1469-0691.12697

[7] Fritzenwanker M, Imirzalioglu C, Herold S, Wagenlehner FM, Zimmer K-P, Chakraborty T. Treatment options for Carbapenem-resistant gram-negative infections. Dtsch Arztebl Int. 2018;115(20–21):345. 29914612 10.3238/arztebl.2018.0345 PMC6172649

[8] Marchetti A, Rossiter R. Economic burden of healthcare-associated infection in US acute care hospitals: Societal perspective. J Med Econ. 2013;16(12):1399–1404. 24024988 10.3111/13696998.2013.842922

[9] Dalton KR, Rock C, Carroll KC, Davis MF. One health in hospitals: How understanding the dynamics of people, animals, and the hospital built-environment can be used to better inform interventions for antimicrobial-resistant gram-positive infections. Antimicrob Resist Infect Control. 2020;9(1):78. 32487220 10.1186/s13756-020-00737-2 PMC7268532

[10] Denton M. Enterobacteriaceae. Int J Antimicrob Agents. 2007;29:S9–S22. 17659212 10.1016/S0924-8579(07)72174-X

[11] Jamrozik E, Selgelid MJ. Invisible epidemics: Ethics and asymptomatic infection. Monash Bioeth Rev. 2020;38(S1):1–16. 10.1007/s40592-020-00123-z PMC7738616 33326062

[12] Gao Y, Chen M, Cai M, Liu K, Wang Y, Zhou C, Chang Z, Zou Q, Xiao S, Cao Y, et al. An analysis of risk factors for Carbapenem-resistant Enterobacteriaceae infection. J Glob Antimicrob Resist. 2022;30:191–198. 35429666 10.1016/j.jgar.2022.04.005

[13] Akturk H, Sutcu M, Somer A, Aydın D, Cihan R, Ozdemir A, Coban A, Ince Z, Citak A, Salman N. Carbapenem-resistant *Klebsiella pneumoniae* colonization in pediatric and neonatal intensive care units: Risk factors for progression to infection. Braz J Infect Dis. 2016;20(2):134–140. 26867474 10.1016/j.bjid.2015.12.004 PMC9427560

[14] World Health Organization. *Health care without avoidable infections: The critical role of infection prevention and control*. World Health Organization; 2016.

[15] Liu Q, Yang J, Zhang J, Zhao F, Feng X, Wang X, Lyu J. Description of clinical characteristics of VAP patients in MIMIC database. Front Pharmacol. 2019;10:62. 30778301 10.3389/fphar.2019.00062 PMC6369200

[16] Lin J, Gu C, Zhang S, Tian L, Ren K, Cao Z, Han X. Sites and causes of infection in patients with sepsis-associated liver dysfunction: A population study from the medical information mart for intensive care III. Med Sci Monit. 2021;27:e928928–e928921. 33638975 10.12659/MSM.928928 PMC7927361

[17] Zhao L, Gao Y, Guo S, Lu X, Yu S, Ge Z, Zhu H, Li Y. Prognosis of patients with sepsis and non-hepatic hyperammonemia: A cohort study. Med Sci Monit. 2020;26:e928573–e928571. 33373333 10.12659/MSM.928573 PMC7777151

[18] Peiffer-Smadja N, Rawson TM, Ahmad R, Buchard A, Georgiou P, Lescure F-X, Birgand G, Holmes AH. Machine learning for clinical decision support in infectious diseases: A narrative review of current applications. Clin Microbiol Infect. 2020;26(5):584–595. 31539636 10.1016/j.cmi.2019.09.009

[19] Hirano Y, Shinmoto K, Okada Y, Suga K, Bombard J, Murahata S, Shrestha M, Ocheja P, Tanaka A. Machine learning approach to predict positive screening of methicillin-resistant *Staphylococcus aureus* during mechanical ventilation using synthetic dataset from MIMIC-IV database. Front Med. 2021;8:694520. 10.3389/fmed.2021.694520 PMC8635043 34869405

[20] Baldominos A, Puello A, Oğul H, Aşuroğlu T, Colomo-Palacios R. Predicting infections using computational intelligence—A systematic review. IEEE Access. 2020;8:31083–31102.

[21] Teodoro D, Lovis C. Empirical mode decomposition and K-nearest embedding vectors for timely analyses of antibiotic resistance trends. PLoS One. 2013;8(4): Article e61180. 23637796 10.1371/journal.pone.0061180 PMC3636283

[22] Teodoro D, Pasche E, Gobeill J, Emonet S, Ruch P, Lovis C. Building a transnational biosurveillance network using semantic web technologies: Requirements, design, and preliminary evaluation. J Med Internet Res. 2012;14(3): Article e2043. 10.2196/jmir.2043 PMC3799609 22642960

[23] Hartvigsen T, Sen C, Brownell S, Teeple E, Kong X, Rundensteiner EA. Early prediction of MRSA infections using electronic health records. In: *HEALTHINF*. Setúbal (Portugal): SciTePress; 2018. p. 156–167.

[24] Jeng S-L, Huang Z-J, Yang D-C, Teng C-H, Wang M-C. Machine learning to predict the development of recurrent urinary tract infection related to single uropathogen, *Escherichia coli*. Sci Rep. 2022;12(1):17216. 36241875 10.1038/s41598-022-18920-3 PMC9568612

[25] Yang D, Xie Z, Xin X, Xue W, Zhang M. A model for predicting nosocomial Carbapenem-resistant *Klebsiella pneumoniae* infection. Biomed Rep. 2016;5(4):501–505. 27699021 10.3892/br.2016.752 PMC5038556

[26] Sen C, Hartvigsen T, Rundensteiner E, Claypool K. Crest-risk prediction for *Clostridium difficile* infection using multimodal data mining. In: *Joint European Conference on Machine Learning and Knowledge Discovery in Databases*. Cham (Germany): Springer; 2017. p. 52–63.

[27] van Niekerk JM, Lokate M, Braakman-Jansen LMA, van Gemert-Pijnen J, Stein A. Spatiotemporal prediction of vancomycin-resistant *Enterococcus* colonisation. BMC Infect Dis. 2022;22(1):1–12. 35057734 10.1186/s12879-022-07043-9 PMC8781237

[28] Çaǧlayan Ç, Barnes SL, Pineles LL, Harris AD, Klein EY. A data-driven framework for identifying intensive care unit admissions colonized with multidrug-resistant organisms. Front Public Health. 2022;10: Article 853757. 35372195 10.3389/fpubh.2022.853757 PMC8968755

[29] Goodman KE, Simner PJ, Klein EY, Kazmi AQ, Gadala A, Toerper MF, Levin S, Tamma PD, Rock C, Cosgrove SE, et al. Predicting probability of perirectal colonization with Carbapenem-resistant Enterobacteriaceae (CRE) and other Carbapenem-resistant organisms (CROs) at hospital unit admission. Infect Control Hosp Epidemiol. 2019;40(5):541–550. 30915928 10.1017/ice.2019.42 PMC6613376

[30] Kawaguchi K, Kaelbling LP, Bengio Y. Generalization in deep learning. arXiv. 2017. 10.48550/arXiv.1710.05468

[31] Si Y, Du J, Li Z, Jiang X, Miller T, Wang F, Zheng WJ, Roberts K. Deep representation learning of patient data from electronic health records (EHR): A systematic review. J Biomed Inform. 2021;115: Article 103671. 33387683 10.1016/j.jbi.2020.103671 PMC11290708

[32] Kipf TN, Welling M. Semi-supervised classification with graph convolutional networks. arXiv. 2016. 10.48550/arXiv.1609.02907

[33] Johnson AE, Pollard TJ, Shen L, Lehman LH, Feng M, Ghassemi M, Moody B, Szolovits P, Anthony Celi L, Mark RG. MIMIC-III, a freely accessible critical care database. Sci data. 2016;3(1):1–9. 10.1038/sdata.2016.35 PMC4878278 27219127

[34] Grover A, Leskovec J. Node2vec: Scalable feature learning for networks. In: *Proceedings of the 22nd ACM SIGKDD International Conference on Knowledge Discovery and Data Mining*. (New York, USA): Association for Computing Machinery; 2016. p. 855–864. 10.1145/2939672.2939754 PMC5108654 27853626

[35] Magiorakos A-P, Srinivasan A, Carey RB, Carmeli Y, Falagas ME, Giske CG, Harbarth S, Hindler JF, Kahlmeter G, Olsson-Liljequist B, et al. Multidrug-resistant, extensively drug-resistant and Pandrug-resistant bacteria: An international expert proposal for interim standard definitions for acquired resistance. Clin Microbiol Infect. 2012;18(3):268–281. 21793988 10.1111/j.1469-0691.2011.03570.x

[36] Schoch CL, Ciufo S, Domrachev M, Hotton CL, Kannan S, Khovanskaya R, Leipe D, Mcveigh R, O’Neill K, Robbertse B, et al. NCBI taxonomy: A comprehensive update on curation, resources and tools. Database (Oxford). 2020;2020:baaa062. 32761142 10.1093/database/baaa062 PMC7408187

[37] Bonten MJ, Gaillard CA, Johanson WG Jr, van Tiel FH, Smeets HG, Van Der Geest S, Stobberingh EE. Colonization in patients receiving and not receiving topical antimicrobial prophylaxis. Am J Respir Crit Care Med. 1994;150(5):1332–1340. 7952561 10.1164/ajrccm.150.5.7952561

[38] Pedregosa F, Varoquaux G, Gramfort A, Michel V, Thirion B, Grisel O, Blondel M, Prettenhofer P, Weiss R, Dubourg V. Scikit-Learn: Machine learning in python. J Mach Learn Res. 2011;12:2825–2830.

[39] Scarselli F, Gori M, Tsoi AC, Hagenbuchner M, Monfardini G. The graph neural network model. IEEE Trans Neural Netw. 2008;20(1):61–80. 19068426 10.1109/TNN.2008.2005605

[40] Battaglia PW, Hamrick JB, Bapst V, Sanchez-Gonzalez A, Zambaldi V, Malinowski M, Tacchetti A, Raposo D, Santoro A, Faulkner R. Relational inductive biases, deep learning, and graph networks. arXiv. 2018. 10.48550/arXiv.1806.01261

[41] Bronstein MM, Bruna J, Cohen T, Veličković P. Geometric deep learning: Grids, groups, graphs, geodesics, and gauges. arXiv. 2021. https://doi.org/10.48550/arXiv.2104.13478

[42] Veličković P, Cucurull G, Casanova A, Romero A, Lio P, Bengio Y. Graph attention networks. arXiv. 2017. 10.48550/arXiv.1710.10903

[43] Hamilton W, Ying Z, Leskovec J. Inductive representation learning on large graphs. Adv Neural Inf Process Syst. 2017.

[44] Lin T-Y, Goyal P, Girshick R, He K, Dollár P. Focal loss for dense object detection. In: *Proceedings of the IEEE International Conference on Computer Vision*. New York (USA): Institute of Electrical and Electronics Engineers (IEEE); 2017. p. 2980–2988.

[45] Loshchilov I, Hutter F. Decoupled weight decay regularization. arXiv. 2017. https://doi.org/10.48550/arXiv.1711.05101

[46] Akiba T, Sano S, Yanase T, Ohta T, Koyama M. Optuna: A next-generation hyperparameter optimization framework. In: *Proceedings of the 25th ACM SIGKDD International Conference on Knowledge Discovery & Data Mining.* New York (USA): Association for Computing Machinery; 2019. p. 2623–2631.

[47] Bergstra J, Bardenet R, Bengio Y, Kégl B. Algorithms for hyper-parameter optimization. Adv Neural Inf Process Syst. 2011.

[48] Cunningham P, Delany SJ. K-nearest neighbour classifiers-a tutorial. ACM Comput Surv (CSUR). 2021;54(6):1–25.

[49] Wright RE. *Logistic regression*. Washington (DC): American Psychological Association; 1995.

[50] Breiman L. Random forests. Mach Learn. 2001;45(1):5–32.

[51] Prokhorenkova L, Gusev G, Vorobev A, Dorogush AV, Gulin A. CatBoost: Unbiased boosting with categorical features. Adv Neural Inf Process Syst. 2018;31.

[52] Mikolov T, Sutskever I, Chen K, Corrado GS, Dean J. Distributed representations of words and phrases and their compositionality. Adv Neural Inf Process Syst. 2013.

[53] Lundberg SM, Lee S-I. A unified approach to interpreting model predictions. Adv Neural Inf Process Syst. 2017.

[54] Harrington RD, Hooton TM. Urinary tract infection risk factors and gender. J Gend Specif Med. 2000;3(8):27–34. 11253265

[55] Vincent J-L. Nosocomial infections in adult intensive-care units. Lancet. 2003;361(9374):2068–2077. 12814731 10.1016/S0140-6736(03)13644-6

[56] Abbas M, Robalo Nunes T, Martischang R, Zingg W, Iten A, Pittet D, Harbarth S. Nosocomial transmission and outbreaks of coronavirus disease 2019: The need to protect both patients and healthcare workers. Antimicrob Resist Infect Control. 2021;10(1):1–13. 33407833 10.1186/s13756-020-00875-7 PMC7787623

[57] Cassini A, Högberg LD, Plachouras D, Quattrocchi A, Hoxha A, Simonsen GS, Colomb-Cotinat M, Kretzschmar ME, Devleesschauwer B, Cecchini M, et al. Attributable deaths and disability-adjusted life-years caused by infections with antibiotic-resistant bacteria in the EU and the European economic area in 2015: A population-level modelling analysis. Lancet Infect Dis. 2019;19(1):56–66. 30409683 10.1016/S1473-3099(18)30605-4 PMC6300481

[58] Boonstra MB, Spijkerman DC, Voor AF, van der Laan RJ, Bode LG, van Vianen W, Klaassen CH, Vos MC, Severin JA. An outbreak of ST307 extended-spectrum beta-lactamase (ESBL)–producing *Klebsiella pneumoniae* in a rehabilitation center: An unusual source and route of transmission. Infect Control Hosp Epidemiol. 2020;41(1):31–36. 31685058 10.1017/ice.2019.304

[59] Dik J-WH, Hendrix R, Poelman R, Niesters HG, Postma MJ, Sinha B, Friedrich AW. Measuring the impact of antimicrobial stewardship programs. Expert Rev Anti-Infect Ther. 2016;14(6):569–575. 27077229 10.1080/14787210.2016.1178064

[60] Patel G, Huprikar S, Factor SH, Jenkins SG, Calfee DP. Outcomes of Carbapenem-resistant *Klebsiella pneumoniae* infection and the impact of antimicrobial and adjunctive therapies. Infect Control Hosp Epidemiol. 2008;29(12):1099–1106. 18973455 10.1086/592412

[61] McHaney-Lindstrom M, Hebert C, Flaherty J, Mangino JE, Moffatt-Bruce S, Root ED. Analysis of intra-hospital transfers and hospital-onset clostridium difficile infection. J Hosp Infect. 2019;102(2):168–169. 30172746 10.1016/j.jhin.2018.08.016 PMC7321831

[62] Uslan DZ, Crane SJ, Steckelberg JM, Cockerill FR, Sauver JLS, Wilson WR, Baddour LM. Age-and sex-associated trends in bloodstream infection: A population-based study in Olmsted County, Minnesota. Arch Int Med. 2007;167(8):834–839. 17452548 10.1001/archinte.167.8.834

[63] Livermore DM, Pearson A. Antibiotic resistance: Location, location, location. Clin Microbiol Infect. 2007;13:7–16. 17488371 10.1111/j.1469-0691.2007.01724.x

[64] Brown PD, Freeman A, Foxman B. Prevalence and predictors of trimethoprim-sulfamethoxazole resistance among uropathogenic *Escherichia coli* isolates in Michigan. Clin Infect Dis. 2002;34(8):1061–1066. 11914994 10.1086/339491

[65] Blanco N, O’Hara LM, Harris AD. Transmission pathways of multidrug-resistant organisms in the hospital setting: A scoping review. Infect Control Hosp Epidemiol. 2019;40(4):447–456. 30837029 10.1017/ice.2018.359 PMC6897300

[66] Hittmeir M, Ekelhart A, Mayer R. Utility and privacy assessments of synthetic data for regression tasks. In: *2019 IEEE International Conference on Big Data (Big Data)*. New York (USA): IEEE; 2019. p. 5763–5772

[67] Hittmeir M, Ekelhart A, Mayer R. On the utility of synthetic data: An empirical evaluation on machine learning tasks. In: *Proceedings of the 14th International Conference on Availability, Reliability and Security*. 2019. p. 1–6.

