# Erratum to “Detection of Patients at Risk of Multidrug-Resistant Enterobacteriaceae Infection Using Graph Neural Networks: A Retrospective Study”

**DOI:** 10.34133/hds.0216

**Published:** 2023-12-16

**Authors:** Racha Gouareb, Alban Bornet, Dimitrios Proios, Sónia Gonçalves Pereira, Douglas Teodoro

**Affiliations:** ^1^ Department of Radiology and Medical Informatics, University of Geneva, Geneva, Switzerland.; ^2^ HES-SO University of Applied Arts Sciences and Arts of Western Switzerland, Geneva, Switzerland.; ^3^ Center for Innovative Care and Health Technology, Polytechnic of Leiria, Leiria, Portugal.; ^4^ Swiss Institute of Bioinformatics, Lausanne, Switzerland.

In the Research Article “Detection of patients at risk of multidrug-resistant Enterobacteriaceae infection using graph neural networks: A retrospective study”, Table 2 was incorrectly duplicated [1]. The correct Table 1 is below, and this has also been updated in the original publication. The publisher apologizes for this error.

**Table 1.** Statistics of the cohort used for model training and evaluation

	Noncolonized (*n* = 267,100)	Colonized (*n* = 7,216)
Sex	116,886 (43.8%)	3,631 (50.3%)
Female		
Male	150,214 (56.2%)	3,585 (49.7%)
Age (years)	35,446 (13.3%)	152 (2.1%)
0–17		
18–25	6,325 (2.4%)	130 (1.8%)
26–45	29,023 (10.9%)	754 (10.4%)
46–65	83,912 (31.4%)	2,436 (33.7%)
66–88	101,629 (38.0%)	3,461 (48.0%)
≥89	10,765 (4.0%)	283 (4.0%)
Reason for admission	34,162 (12.8%)	122 (1.7%)
Newborn		
Pneumonia	6,736 (2.5%)	140 (1.9%)
Sepsis	4,603 (1.7%)	222 (3.1%)
Coronary artery disease	4,449 (1.7%)	52 (0.7%)
Congestive heart failure	4,236 (1.6%)	121 (1.7%)
Chest pain	3,905 (1.5%)	75 (1.0%)
Other	209,009 (78.2%)	6,484 (89.9%)
Resistance profile	–	3,288 (45.6%)
AMS		
AMR	–	3,928 (54.4%)
MDR	–	2,099 (29.1%)
Length of stay (days)	57,511 (21.5%)	407 (5.6%)
0–4		
5–10	105,531 (39.5%)	1,566 (21.7%)
11–50	93,651 (35.1%)	4,284 (59.4%)
51–100	8,183 (3.1%)	738 (10.2%)
≥100	2,224 (0.8%)	221 (3.1%)
Specimen	–	2,954 (40.9%)
Urine		
Sputum	–	1,944 (26.9%)
Blood	–	934 (12.9%)
Skin	–	483 (6.7%)
Bronchoalveolar lavage	–	288 (4.1%)
Other	–	613 (8.5%)
Enterobacteriaceae species	–	3,385 (47.0%)
*E. coli*		
*K. pneumoniae*	–	2,142 (29.7%)
*E. cloacae*	–	939 (13.0%)
*K. oxytoca*	–	410 (5.7%)
*C. freundii*	–	191 (2.6%)
*C. koseri*	–	107 (1.5%)
Other	–	42 (0.5%)
