# Supplementary material for: Detection of Patients at Risk of Multidrug-Resistant Enterobacteriaceae Infection Using Graph Neural Networks: A Retrospective Study
Source: Health Data Sci. 2023 Nov 20;3:0099. doi: 10.34133/hds.0099 (PMC10904075; doi:10.34133/hds.0099)
Supplement: Supplementary 1 — Table S1 [file hds.0099.f1.docx]

Table S1: Performance of GNNs using different layer types. Model hyper-parameters were tuned using the same procedure as for generating results in Table 2 but keeping the layer type fixed.

| Layer Type | Data Balance | Setting | Links | Accuracy (%) | Sensitivity (%) | Specificity  (%) | AUROC (%)  (95% CI) |
| --- | --- | --- | --- | --- | --- | --- | --- |
|  | | | | | | | |
| GCN | non | inductive | all | 55.46 | 93.89 | 54.42 | 85.79 (85.04-86.63) |
| GCN | non | inductive | in-ward | 84.59 | 74.32 | 84.87 | 88.14 (87.43-88.86) |
| GCN | non | inductive | out-ward | 63.20 | 90.84 | 62.45 | 84.96 (84.32-85.54) |
|  | | | | | | | |
| GCN | non | transductive | all | 83.17 | 83.69 | 83.15 | 90.12 (89.46-90.94) |
| GCN | non | transductive | in-ward | 77.08 | 92.23 | 76.67 | 92.32 (91.80-92.94) |
| GCN | non | transductive | out-ward | 70.79 | 83.83 | 70.43 | 85.31 (84.31-86.13) |
|  | | | | | | | |
| GAT | non | inductive | all | 79.34 | 76.34 | 79.42 | 85.92 (85.29-86.70) |
| GAT | non | inductive | in-ward | 83.76 | 75.92 | 83.97 | 87.60 (86.82-88.38) |
| GAT | non | inductive | out-ward | 83.76 | 68.15 | 84.18 | 85.15 (84.42-86.11) |
|  | | | | | | | |
| GAT | non | transductive | all | 85.86 | 79.60 | 86.03 | 90.24 (89.55-90.86) |
| GAT | non | transductive | in-ward | 93.52 | 69.88 | 94.16 | 92.07 (91.45-92.76) |
| GAT | non | transductive | out-ward | 90.70 | 57.46 | 91.59 | 86.81 (85.86-87.67) |
|  | | | | | | | |
| GraphSage | non | inductive | all | 84.23 | 78.21 | 84.39 | 89.67 (88.87-90.35) |
| GraphSage | non | inductive | in-ward | 82.74 | 83.00 | 82.73 | 91.23 (90.61-91.85) |
| GraphSage | non | inductive | out-ward | 84.89 | 71.55 | 85.25 | 87.90 (87.14-88.67) |
|  | | | | | | | |
| GraphSage | non | transductive | all | 92.36 | 80.50 | 92.68 | 94.07 (93.59-94.60) |
| GraphSage | non | transductive | in-ward | 96.18 | 80.57 | 96.60 | 96.13 (95.63-96.60) |
| GraphSage | non | transductive | out-ward | 83.55 | 79.81 | 83.65 | 89.36 (88.62-89.98) |
